# Neurochemical Correlates of Brain Atrophy in Fibromyalgia Syndrome: A Magnetic Resonance Spectroscopy and Cortical Thickness Study

**DOI:** 10.3390/brainsci10060395

**Published:** 2020-06-20

**Authors:** Paola Feraco, Salvatore Nigro, Luca Passamonti, Alessandro Grecucci, Maria Eugenia Caligiuri, Cesare Gagliardo, Antonella Bacci

**Affiliations:** 1 Neuroradiology Unit, S. Chiara Hospital, Largo Medaglie d’oro 9, 38122 Trento, Italy; 2 Department of Experimental, Diagnostic and Specialty Medicine (DIMES), University of Bologna, Via San Giacomo 14, 40100 Bologna, Italy; 3 Neuroscience Research Center, University “Magna Graecia” of Catanzaro, Viale Europa, 88100 Catanzaro, Italy; salvatoreangelo.nigro@gmail.com (S.N.); mariaeugenia.caligiuri@gmail.com (M.E.C.); 4 Department of Clinical Neurosciences, University of Cambridge, Cambridge CB2 1TN, UK; lp337@medschl.cam.ac.uk; 5 Istituto di Bioimmagini e Fisiologia Molecolare (IBFM), CNR, Via Fratelli Cervi, Segrate, 93-20090 Milan, Italy; 6 Department of Psychology and Cognitive Sciences, University of Trento, Corso Bettini, 84 I-38068 Rovereto, Italy; alessandro.grecucci@unitn.it; 7 Section of Radiological Sciences, Department of Biomedicine, Neurosciences and Advanced Diagnostics, University of Palermo, Via Del Vespro, 90127 Palermo, Italy; cesare.gagliardo@unipa.it; 8 Neuroradiology Unit, IRCSS Istituto Delle Scienze Neurologiche di Bologna, Via Altura 3, 40100 Bologna, Italy; antonella.bacci@isnb.it

**Keywords:** fibromyalgia, glutamate excitotoxicity, cortical thickness, brain MRI, chronic pain, MR spectroscopy

## Abstract

(1) Background: Recently, a series of clinical neuroimaging studies on fibromyalgia (FM) have shown a reduction in cortical volume and abnormally high glutamate (Glu) and glutamate + glutamine (Glx) levels in regions associated with pain modulation. However, it remains unclear whether the volumetric decreases and increased Glu levels in FM are related each other. We hypothesized that higher Glu levels are related to decreases in cortical thickness (CT) and volume in FM patients. (2) Methods: Twelve females with FM and 12 matched healthy controls participated in a session of combined 3.0 Tesla structural magnetic resonance imaging (MRI) and single-voxel MR spectroscopy focused on the thalami and ventrolateral prefrontal cortices (VLPFC). The thickness of the cortical and subcortical gray matter structures and the Glu/Cr and Glx/Cr ratios were estimated. Statistics included an independent *t*-test and Spearman’s test. (3) Results: The Glu/Cr ratio of the left VLPFC was negatively related to the CT of the left inferior frontal gyrus (pars opercularis (*p* = 0.01; *r* = −0.75) and triangularis (*p* = 0.01; *r* = −0.70)). Moreover, the Glx/Cr ratio of the left VLPFC was negatively related to the CT of the left middle anterior cingulate gyrus (*p* = 0.003; *r* = −0.81). Significantly lower CTs in FM were detected in subparts of the cingulate gyrus on both sides and in the right inferior occipital gyrus (*p <* 0.001). (4) Conclusions: Our findings are in line with previous observations that high glutamate levels can be related, in a concentration-dependent manner, to the morphological atrophy described in FM patients.

## 1. Introduction

Fibromyalgia (FM) is a disease characterized by chronic, widespread non-articular pain and diffuse pain to light palpation without an identifiable peripheral pathology [1]. The individual nature and the extreme heterogeneity of FM symptoms have led to a discussion regarding the nature of this condition. However, there is general consensus that it represents a type of central sensitization that results in chronic pain [2]. In other words, intense and protracted pain processing can give rise to neuroplastic changes that perpetuate central sensitization [3,4]. Over the past decade, a few proton magnetic resonance spectroscopy (H-MRS) studies have demonstrated changes in cerebral metabolites in FM patients [5]. These results have reinforced the hypothesis of a neural dysfunction underlying pain processing. In particular, a mismatch between excitatory (glutamate/glutamine) and inhibitory neurotransmitters (gamma-aminobutyric acid, GABA) has been posited as a central pathophysiological mechanism in FM [6]. These alterations seem to have an effect both on lowered pain thresholds and the genesis of chronic pain. Higher glutamate (Glu) and Glu + glutamine (Glx) levels in patients with FM have been consistently found and reported to positively correlate with clinical pain scores [5]. Comparative studies have also shown that the accumulation of extracellular Glu is toxic to neurons, leading to neuronal damage [7]. In particular, abnormally high levels of Glu, resulting from increased release and/or decreased synaptic removal, can cause, in a dose-dependent manner [8], the degeneration and death of neurons [9,10]. A separate line of research has also shown that FM patients display volume reductions in a series of cortical and subcortical regions that have been collectively linked to pain processing and modulation. These regions include the anterior and posterior cingulate cortex, nucleus accumbens, amygdala, insula, caudate nucleus, prefrontal areas and occipital cortex [11]. However, the potential association between brain structural changes and Glu levels in FM has not been studied yet. This is surprising given the pre-clinical evidence showing that the Glu concentration is toxic and can lead to neuronal degeneration [7,8,9,10]. To address this issue, we recruited a sample of patients with FM and compared them with age- and sex-matched healthy controls (HC), in terms of cortical volumetric alterations and Glu metabolic changes. Next, and more importantly, we assessed if structural brain changes are related to Glu levels within the group of patients alone. We hypothesized that higher within-patient levels of Glu related to greater neuronal loss and damage, as assessed by sub-cortical volumetric measures and cortical thickness (CT) indices derived from structural magnetic resonance imaging (MRI).

## 2. Materials and Methods

### 2.1. Subjects

The institutional review board of our hospital approved this retrospective study (Etic Committee: APSS, protocol 4879/2019). All participants provided written informed consent prior to any study procedures. Twelve patients (11 F, 1 M; 30–54 years; mean age 43.2 years) and 12 healthy controls (HC) matched for sex and age (28–56 years; mean age 41.3 years) were recruited (see Table 1). The FM patients met the 1990 American College of Rheumatology criteria for diagnosis of FM [1], without any comorbidity (other rheumatologic diseases and/or central nervous system pathologies). All patients studied where adopted from our previous report [12], but the present is a new investigation of the relationship between the structural data and metabolites levels which was conducted concurrently, but not previously reported. In particular, all patients suspended pharmacologic therapies that are known to affect brain function a week before the study to avoid possible biases, with the exception of acetaminophen, which was permitted to control pain. None of the control subjects took drugs or illicit substances that affected cognitive functions or brain metabolites.

### 2.2. Brain Imaging and Spectroscopy

**Figure 1:**
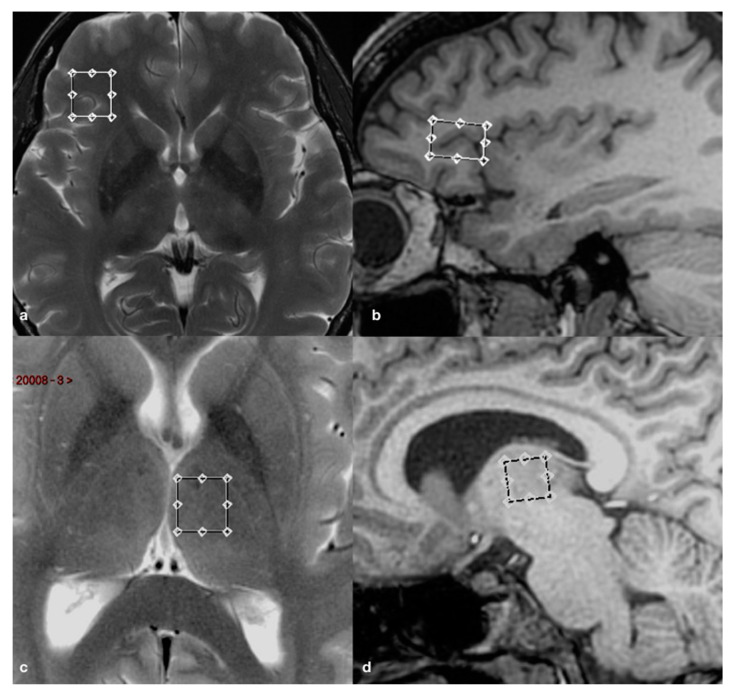
Axial T2-weighted fast spin echo (FSE) and sagittal 3D fast spoiled gradient recalled (FSPGR) images showing voxels of interest (VOIs) placement in the right ventrolateral prefrontal cortex (VLPFC) (**a**,**b**) and left thalamus (**c**,**d**).

All MR imaging were performed by using a 3.0 Tesla whole-body scanner (Signa EXCITE; GE Healthcare, Milwaukee, Wisconsin) equipped with an 8-channel standard phased array head coil. T2-weighted fast spin echo (FSE) sequences were acquired in the axial and coronal planes, while a 3D T1-weighed fast spoiled gradient recalled echo (FSPGR), with an isotropic voxel, was obtained with reconstructions on the axial and coronal planes. A point resolved spectroscopy (PRESS) pulse sequence (TR 2000 ms; TE 35 ms; 128 acquisitions) was used to generate the spectra of the volumes of interest (VOI; 1.8 and 2.1 cm^3^). The voxels of interest (VOIs) were placed bilaterally on the thalamus and ventrolateral prefrontal cortex (VLPFC), respectively, using the 3D FSPGR and FSE structural scans as references to avoid sampling contaminations by bone, vascular and cerebrospinal fluid (CSF) structures (Figure 1). 

Detail on spectra acquisition and processing techniques were previously described [12]. The raw data from each spectrum were evaluated with LCModel 6.1 (Stephen Provencher, Oakville, Ontario, Canada), a user-independent fitting routine based on a library of model spectra of all individual metabolites [13]. The data evaluation comprised a correction of the spectroscopic time domain data for residual eddy current effects. Values for Glu, Gln and Glx (glutamate + glutamine) were calculated as ratios to an internal standard, the creatine (Cr) level, in the form Glu/Cr, Gln/Cr and Glx/Cr. Thus, we did not correct for cerebrospinal fluid (CSF) levels.

### 2.3. Cortical and Subcortical Segmentations

The FreeSurfer image analysis suite (version 5.3.0) (http://surfer.nmr.mgh.harvard.edu/) was used to extract the cortical and subcortical thickness estimations. The detailed procedure for surface reconstruction with FreeSurfer has been previously described and validated elsewhere [14,15,16]. In brief, the pipeline involves the removal of non-brain tissue, automated Talaraich transformation, segmentation of white and grey matter (GM), tessellation of the grey/white matter boundary, automated correction of the topology defects, surface deformation to form the grey/white matter boundary and grey/cerebrospinal fluid boundary, and parcellation of the cerebral cortex. Next, cortical thickness (CT) estimates were calculated as the distance between the grey/white matter border and the pial surface at each vertex. Finally, mean CT values were extracted based on the Destrieux et al. atlas [17]. Subcortical volumes were calculated with FreeSurfer’s automated procedure for volumetric measures [18]. Each voxel in the normalized brain volume was assigned to one label using a probabilistic atlas obtained from a manually labeled training set. All processed images were visually inspected by a neuroradiologist with 15 years’ experience in conventional and advanced neuroimaging techniques, to confirm proper segmentation.

In the present study, we focused our attention first on the pain-processing regions studied with the spectroscopy technique (VLPFCs—which are located in the inferior frontal gyrus—and thalami) and previously reported to be involved in FM and chronic pain, including: cingulate cortex subparts, insular cortex, temporal cortex and occipital cortex. The analysis of subcortical structures included the bilateral caudate, putamen, thalamus and brainstem.

### 2.4. Statistical Analysis

All statistical analyses were run in R v. 3.6.1 (https://www.r-project.org). Differences in the CT and volumes of subcortical structures between the two groups were compared using an independent *t*-test. In the group of patients, Spearman’s rho was calculated to assess the relationship between the cerebral structural measurements of the VLPFCs, thalami and their corresponding Glu/Cr, Gln/Cr and Glx/Cr ratios. Since high levels of Glx/Cr were previously reported on the cingulate cortex of FM patients [5], which is one of the key regions of pain processing, we explored also the relationship between its concentrations in the sampled regions and cingulate gyri thickness.

Analyses assessing these correlations were controlled for age and intracranial volume. Considering that our a priori primary aim was to explore the effect of Glu/Cr or Glx/Cr on neuronal atrophy of the defined regions involved in pain processing, for all pairwise comparisons and correlation analyses, the level of significance was set to *p* ≤ 0.01, with no adjustment for multiple comparisons.

## 3. Results

### 3.1. Relationship between Metabolites and Brain Structural Data

The results of the significant relationships between the structural data and metabolites are summarized in the Table 2. 

**Figure 2:**
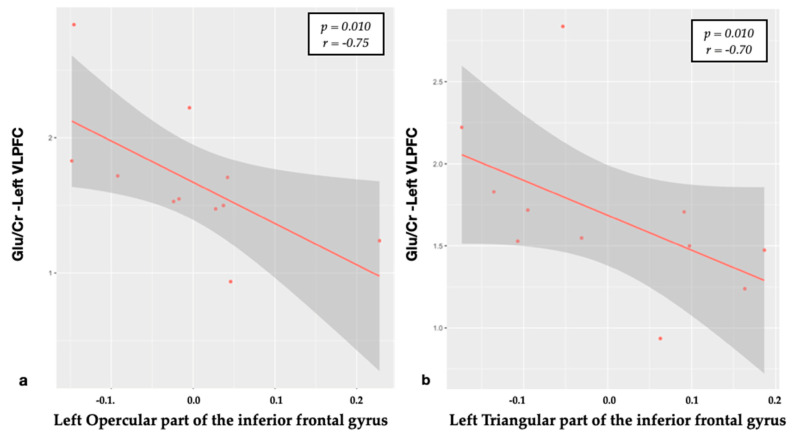
The Spearman’s correlation of Glu/Cr in the left VLPFC and cortical thickness of the opercular (**a**) and triangular (**b**) parts of the left inferior frontal gyrus (residuals).

Frontal regions: on the left side, the Glu/Cr ratio was negatively related to the CT of the inferior frontal gyrus in its pars opercularis and triangularis (*p* = 0.01) (Figure 2). Moreover, the Glx/Cr ratio of the left VLPFC was negatively related to the CT of the left middle anterior cingulate gyrus (mACG) (*p* = 0.003).

**Figure 3:**
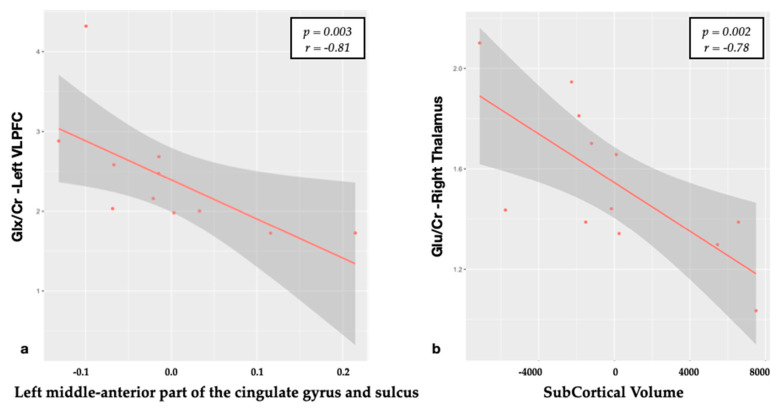
Correlation between metabolite ratios and cortical thickness. (**a**) The Spearman’s correlation of Glx/Cr in the left VLPFC and cortical thickness of left middle anterior part of the cingulate gyrus (residuals); (**b**) Spearman’s correlation of Glu/Cr in the right thalamus and subcortical gray matter volume (residuals).

Thalamic regions: a significant negative relationship was found between the Glu/Cr levels in the right thalamus with the subcortical GM volume (*p* = 0.002; *r* = −0.78) (Figure 3).

### 3.2. Structural Differences and Clinical Relationships

**Figure 4:**
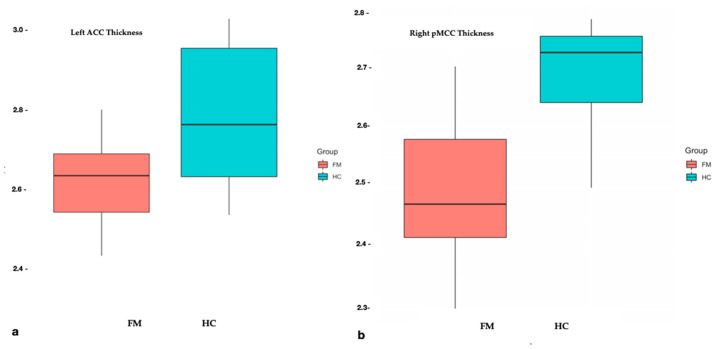
Cortical Thickness differences in cingulate gyrus subparts between fibromyalgia (FM) patients (red boxes) and healthy controls (blue boxes). On each box, the horizontal line is the median and the edges of the box are the 25th and 75th percentiles. The *p*-value was determined by the two-tailed *t*-test for independent samples. (**a**) Left anterior cingulate gyrus; (**b**) middle posterior part of the right cingulate gyrus.

As expected, the CT analysis revealed several regions of significant lower regional values in FM patients compared with HC. These regions included, the left anterior cingulate gyrus (ACG), the right mid-posterior cingulate gyrus (pMCG), and right inferior occipital gyrus (Figure 4). The *p*-values of significant differences between the patients and HC are summarized in the Table 3.

## 4. Discussion

As expected, we found a negative association between Glu levels and CT in the corresponding brain region. In particular, higher left VLPFC Glu/Cr concentrations, previously reported in the same group of patients [12], related in a negative way to the CT values of the inferior frontal gyrus of the same side. This corroborates the hypothesis that the previously detected neurochemical and structural abnormalities reported in FM are related to each other and can synergistically contribute to the pathophysiology of FM. Although we assessed the ratios of metabolites on Cr, we suppose that the relationships between metabolites and CT are driven by increased levels of Glu and Glx, respectively, and not by a reduction in Cr because the absolute Cr levels were homogeneous among patients, as we previously found [12].

The VLPFC, part of the prefrontal cortex, is located on the inferior frontal gyrus, being attributed to the anatomical structures of Brodmann’s areas 47, 45 and 44, anatomically defined as the orbitalis, triangularis and opercularis part, respectively. Although we did not find significant CT differences at the inferior frontal gyrus between groups, we interestingly detected negative relationships between the Glu/Cr levels revealed on the left VLPFC and its subregions (opercularis and triangularis subparts) of the same side (Figure 2). Interestingly, although we also detected higher Glu/Cr levels within the right VLPFC, no significant relations were found with the CT on the corresponding region. The different results of VLPFCs, although may be related to the small patient sample explored, could be also explained by a possible different response of both areas to the chronic pain [19,20].

Moreover, consistent with previous studies, we confirmed the implication of the cingulate cortex in FM. We found a reduction in the CT of the left anterior cingulate gyrus (ACG). Indeed, a reduction in the cortical GM volume in multiple subparts of the CG was widely reported [21,22,23,24], in association with the pain score. In particular, the whole rostral ACG (defined as the totality of anterior CG and middle CG) is considered to be one of the key regions implicated in FM disease. In HC, together with the precuneus, occipital cortex [21] and amygdala, the AC cortex is considered responsible for the descending control of pain. FM patients failed to activate it [25], showing also a decreased connectivity in this pain descending inhibitory network during resting state studies in a default model network [21,26]. In addition, we detected a significant decrease in the right middle posterior cingulate gyrus (pMCG) in FM subjects (*p* = 0.0001). This region is related to orienting the body toward nocuous stimuli, the non-affective component of pain processing, and contributes to the retraction reaction from pain [27]. Moreover, the pMCG has been reported to be involved in several chronic somatic pain disorders [28].

Moreover, although our spectra were not located in the cingulate gyrus, we found a strong negative relationship between Glx/Cr in the left VLPFC and the left aMCG (Figure 3). Relationships of N-acetylaspartate, Glu and myo-inositol in the ACG to the physiology of pain and treatment with morphine were described [29], but patients suffering from chronic pain displayed only high levels of Glx and Glx/Cr both in anterior than posterior CG [30,31]. Although there were no significant differences in the CT between patients and HC (*p* = 0.014) in the left aMCG, the strong negative relation we found with Glx/Cr (*p* = 0.003) could suggest that the toxic effect of high Glu concentrations impacts also on the other regions implicated in pain processing. From a functional standpoint, it represents a hub, where information about pain could be linked to motor centres responsible for expressing emotion on the face and coordinating aversively motivated instrumental behaviours [32]. However, due to the small patient sample and the not-perfect correspondence between the VOI and the region, further studies should confirm these data. 

Interestingly, no correlations were detected between thalami volumes and Glu/Cr at that level. Indeed, in our previous report, we did not find differences between groups in metabolites and their ratios at the thalamus level, strengthening the opinion that Glu could be toxic only at abnormally high concentrations. On the other hand, we found a relation between Glu/Cr in the right thalamus with the total subcortical gray volume. This could reflect how Glu impacts on the overall subcortical regions but not directly on thalami. Further studies, using a multi-voxel spectroscopy approach, could better evaluate this hypothesis.

However, there is strong a priori evidence that Glu excesses can lead to neuronal dysfunction and cell loss, so the relationship we found in this study is interesting and can shed new light into the pathophysiology of FM. In pharmacological and cellular models, all Glu agonists (ionotropic and metabotropic) cause neuronal apoptosis in a dose-dependent manner [8]. Moreover, abnormally high levels of Glu, resulting from increased release and/or decreased removal at the synapses, lead to degeneration and neuronal death [9,10]. The neurotoxic effects of excessive Glu receptor activation have been widely described also in many other conditions including epilepsy, stroke, traumatic brain and spinal cord injury and amyotrophic lateral sclerosis [33]. Glu is the primary excitatory neurotransmitter in the brain, and it is found in approximately 60% of neurons. It is known that synaptic Glu is taken up by astrocytes, where it is converted to glutamine and transported to neurons for the production of Glu or GABA [34]. The implication of glutamatergic neurotransmission in pain processing has been proposed. Indeed, the development of neuropathic pain in preclinical models is thought to be a result of central sensitization, or central plasticity, involving both ionotropic and metabotropic glutamate receptors [4]. Higher Glx and Glu levels within the brain of FM patients have been reported by different groups in several regions, including the amygdala, the posterior cingulate, VLPFC, posterior insula and hippocampus [5]. Moreover, volumetric reductions in these brain regions in FM have been largely described in structural MRI studies [35], but without exploring the metabolites concentrations. However, the Glu elevation does appear specific. Indeed, not every region examined presented this finding. In particular, no elevations have been detected in the anterior insula [36] or the prefrontal cortex [30]. Thus, a topographic distribution of a higher Glu level could be responsible for the atrophic pattern described in this condition playing a role in chronic pain symptoms through neuroplastic changes as detected in animal models [4].

Considering H-MRS and structural studies performed in the same group of FM patients, only a sub-part of a study explored the correlation between Glx changes in the anterior and posterior insula and GM changes after pregabalin administration [37], without finding a significant relationship. Interestingly, the same study found GM reduction in the bilateral insula after pregabalin administration, that was also associated with concomitant reductions in connectivity to the default mode network, and with reduced clinical pain. They speculated that the decreased GM volumes were due to the decreased Glu synapse number. Relative to controls, patients with FM also showed abnormalities in many brain areas as in previous structural neuroimaging studies [11,20,30]. Brain plasticity in the form of increased/decreased GM has been observed in FM [11]. Ceko et al. demonstrated how fibromyalgia interacts with age to produce distinct patterns of gray matter differences, which specifically increases in younger and decreases in older patients, suggesting that the brain structure and function shift from being adaptive in younger to being maladaptive in older patients [38]. Hence, to test the impact of the glutamatergic system on CT and subcortical volumes, we controlled the results for age and intracranial volume. Considering other cortical regions, we found a strong reduction in the right inferior occipital gyrus in the FM group. This result is consistent with the literature, where indeed these regions were previously reported to be altered in the pain-processing network in FM patients [39,40]. 

### Limitations

Our empirical data analysis study has several limitations. First of all, the small group sample size may limit the generalizability of our findings. However, the groups were matched for sex and age, and the cohort is similar to those used in studies of other advanced neuroimaging techniques in FM. Due to the retrospective design of the study (enrolment in 2010), patients were selected using the 1990 ACR criteria for the diagnosis of FM. Further studies should select patients using more recently revised diagnostic criteria carrying a higher specificity for FM diagnosis and chronic widespread pain [41,42] that may be reflected by changes in glutamatergic neurosignaling. We also did not collect information on depression and anxiety symptoms, which may be a potential confounder in any findings related to the role of Glu and regional thickness [40,43]. Considering the spectra, one of the limits is the relatively large size of the H-MRS voxels, but this is required to achieve an adequate signal-to-noise ratio for the quantification of Glu and Glx using the PRESS technique at a 3T field strength. Such large voxels inevitably contain substantial quantities of GM in which the majority of glutamatergic activity occurs, white matter that has less Glu and CSF which contains insignificant amounts of Glu. Moreover, this technique does not differentiate between intracellular and extracellular components, indeed its coediting is limited to the macromolecular signal. However, this study was based on prior observations of a strong association between Glu concentrations and brain atrophy and we therefore interpret our observations as representing actual Glu levels. Indeed, this study provides the first (to our knowledge) promising in vivo evidence of a direct negative relationship between glutamate and regional CT in FM. Therefore, future studies with larger sample sizes are needed to improve the statistical power and verify these observations. 

## 5. Conclusions

Our study is the first, to our knowledge, to show a negative correlation between Glu/Cr levels and brain regional structural changes in FM. Although these results are far from conclusive, they do support a role for Glu in the pathophysiology of FM. Prospective studies with larger sample sizes are needed to confirm these results and to validate this method. We anticipate that clinical research should investigate these aspects of FM in order to better understand the long-term effects of pain on the central nervous system to investigate new therapeutic options. In particular, the development of new therapeutic approaches that aim to limit the effect of Glu-mediated neurotoxicity, thus mediating the clinical severity and progression of FM, is desirable in the near future.

## Figures and Tables

**Table 1:** Demographic and clinical data of twelve patients (Pt) suffering from fibromyalgia and twelve healthy controls (HC).

No. of HC	Age, Year	Sex	No. of Pt	AGE, Year	Sex	Onset, Year	Duration, Year	TP	VAS	FIQ
1	33	F	1	34	F	30	4	11	47	48.4
2	54	F	2	54	F	45	9	18	87	83.15
3	41	F	3	44	F	40	4	18	36	38
4	45	F	4	45	F	42	3	18	70	67.86
5	40	F	5	39	F	36	3	16	50	44
6	48	F	6	49	F	47	1.5	16	80	69.77
7	49	F	7	49	F	42	7	12	85	61
8	45	F	8	45	F	44	1	18	90	74.21
9	41	F	9	39	F	30	9	11	54	58
10	50	F	10	53	F	50	3	12	54	37
11	49	F	11	48	F	47	1	14	60	47
12	52	M	12	41	M	35	6	18	90	80.44

TP (number of tender Points); VAS (visual analogic scale); FIQ (Fibromyalgia Impact Questionnaire).

**Table 2:** Association between metabolites’ ratio values, cortical thickness and subcortical volumes.

	Metabolite	rho	*p*-Value
*Left VLPFC*	-	-	-
Opercular part of the inferior frontal gyrus CT	Glu/Cr	−0.75	0.010
Triangular part of the inferior frontal gyrus CT	Glu/Cr	−0.70	0.010
Middle anterior part of the cingulate gyrus CT	Glx/Cr	−0.81	0.003
*Right thalamus*	-	-	-
SubCortGrayVol	Glu/Cr	−0.78	0.002

**Table 3:** The mean and standard deviation (sd) of the variables for cortical thickness, separately for controls and FM patients. Significant *p*-value were considered for *p* ≤ 0.01.

Cortical Regions	Controls (Mean mm^2^ ± SD)	FM Patients (Mean ± SD)	*p*-Value
Left hemisphere			
Anterior part of the cingulate gyrus and sulcus	2.78 ± 0.17	2.61 ± 0.14	0.007
Right hemisphere			
Inferior occipital gyrus and sulcus	2.66 ± 0.17	2.46 ± 0.19	0.009
Middle posterior part of the cingulate gyrus and sulcus	2.68 ± 0.09	2.49 ± 0.12	<0.001
